# An evaluation of three-dimensional facial changes after surgically assisted rapid maxillary expansion (SARME): an observational study

**DOI:** 10.1186/s12903-022-02179-1

**Published:** 2022-05-02

**Authors:** Jurij Zupan, Nataša Ihan Hren, Miha Verdenik

**Affiliations:** 1 Faculty of Dental Medicine, Vrazov trg 2, 1000 Ljubljana, Slovenia; 2 Faculty of Medicine, Department of Maxillofacial and Oral Surgery, Vrazov trg 2, 1000 Ljubljana, Slovenia; 3 Clinical Department of Maxillofacial and Oral Surgery, University Medical Centre Ljubljana, UKCLJ, Zaloska cesta 2, 1000 Ljubljana, Slovenia

**Keywords:** Facial scan, 3D scanning, Facial soft tissue, Maxillary expansion, Maxillary osteotomy

## Abstract

**Background:**

The abnormal facial features in maxillary transverse deficiency (MTD) are minimal and limited to a deficiency of the middle facial third, narrow nares and nasal base, and deepened nasolabial folds. The surgical expansion of the narrow maxilla has most obvious effects on widening of the maxillary dental arch and expansion of the maxillary and palatal structures in the transverse plane, however sagittal changes also occurs. The purpose of this observational study was to evaluate the three-dimensional (3D) facial soft tissue changes following surgically assisted rapid maxillary expansion (SARME).

**Methods:**

In 15 skeletally mature patients with severe maxillary transverse deficiency, the planned maxillary expansion (on average 8.8 mm ± 2.3 mm) was achieved with a bone-borne palatal distractor. The 3D optical scans of the facial surface were obtained before and six months after SARME. In the first part, we defined different anatomical landmarks on both scans and compared cephalometric measurements. In the second part, we registered both 3D scans in the same workplace using the regional best-fit method (forehead, supraorbital and nasal root regions were selected for the superimposition) and conducted surface analysis.

**Results:**

The largest differences between the pre- and post-operation scans were observed in the paranasal and cheek area (1.4 ± 1.0 mm). Significant differences occurred for an increased nasal width, a decreased upper-face height with an unchanged lower height, an increased vertical philtrum height and an increased nasolabial angle. A significant increase in the facial profile angle was also observed, resulting in an increased facial convexity and anterior displacement of the upper-lip area.

**Conclusions:**

The widening of the nose and increased projection in the cheek and paranasal area in the lateral direction after maxillary expansion were confirmed; moreover, facial convexity increases, reflecting the underlying advancement of the maxilla.

## Background

Maxillary transverse deficiency (MTD) mostly with posterior crossbite and with an intermolar distance of less than 31 mm [1] affects both the function and the appearance of the patient’s orofacial region [2]. It can be isolated or combined with other sagittal or vertical dento-facial deformities that clinically mask the anomalies in the transverse dimension [3]. The abnormal facial features in MTD are minimal and limited to a deficiency of the middle facial third, narrow nares and nasal base, and deepened nasolabial folds [2]. However, an intra-oral examination can reveal a uni- or bilateral posterior cross bite, dental crowding and a narrow and high palatal vault [2]. These abnormalities can also be associated with functional impairments [4, 5].

The transverse expansion of the narrow maxilla by opening the mid-palatal suture was first reported by Angell [6] and rapid maxillary expansion is nowadays a generally accepted method in growing individuals. After the ossification of the mid-palatal suture, which is reported to happen during adolescence (14–15 years of age [7], until the end of adolescence at 18 years of age [8] or even later [9], managing severe MTD requires a correction with surgery. Surgically assisted rapid maxillary expansion (SARME), which combines orthodontics and surgical procedures, is now a widely used procedure for the correction of severe MTD in skeletally mature patients.

Various studies have evaluated immediate and long-term dental, skeletal, periodontal, nasal airway, and facial soft-tissue changes following SARME [10–16]. The most obvious effects of SARME are widening of the maxillary dental arch and expansion of the maxillary and palatal structures in the transverse plane [17]. Facial changes following SARME reflect the underlying dento-skeletal movements. The most frequently reported findings are widening of the nose and an increased projection of the cheek area in the lateral direction [17]. A slight retro-positioning of the upper lip [18] and an anterio-inferior displacement of the whole naso-maxillary complex after SARME were also reported [16].

SARME has a well-known effect on the facial skeletal framework and, consequently, affects the facial (a)esthetic appearance of the face, which should be considered during diagnostics, treatment planning and evaluation. Previous studies evaluating soft-tissue changes after SARME focused mostly on the transvers facial changes and areas where the underlying bones had been expanded [10, 12, 16, 18, 19]. 3D imaging modalities, such as cone-beam computed tomography or stereometric surface imaging, are rapidly replacing conventional 2D technologies [17]. Therefore, we decided to use 3D facial imaging to assess the facial changes, including sagittal changes after SARME, and to evaluate the soft-to-hard tissue ratios for maxillary expansion. The two main hypothesis were that with maxillary distraction also anterior movement of maxilla happen and that changes affects the whole face (lower and upper facial third).

## Methods

We calculated the sample size with setting effect size of 1 (mean divided by SD) and calculated only 11 pairs would be needed. We hypothesized effect size of this magnitude would be expected in at least some of parameters. To be on the safe side we choose a sample of 15 orthodontic patients (8 females, 7 males) with an transverse discrepancy between the maxilla and mandible greater than 7 mm. The median age of the patients at the time of the surgery was 26.0 ± 9.0 years. Each patient’s protocol included information about their sex, age, height, body mass and MTD. Due to the patient age and MTD the SARME was decided as a first step in orthodonthic therapy. Exlusion criteria were history and presentation of any congenital dentofacial deformity and facial asymmetries. The severity of the deformity was defined by orthodontist after standard diagnostics; clinical examination, x-ray and photography analysis and mostly using dental study casts and measuring the transversal distance at the level of the canine and first molar teeth and comparing this to the lower arch. The amount of needed trans-palatal expansion was calculated as an average of the two measured transverse discrepancy levels, and it was found to be 8.8 (± 2.3) millimetres. This study was approved by the Slovenian National Medical Ethics Committee (conformation number 0120-303/2017) and was conducted as prospective observational study in compliance with the Helsinki Declaration and the STROBE statement guidelines. All the patients also signed informed consent.

**Fig. 1:**
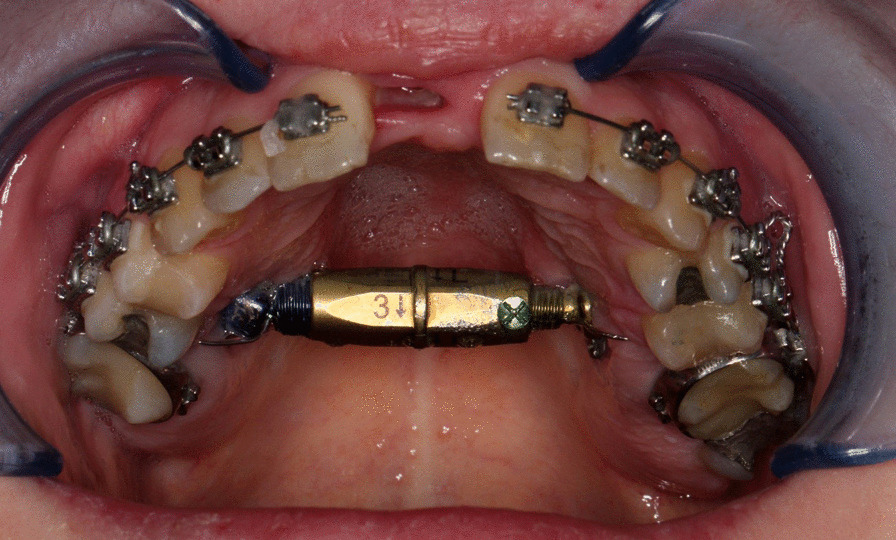
The Synthes transpalatinal distractor we use at our clinic. The image presents the distractor and the site of site of its placement during operation

After initial orthodonthic intervention with fixed orthodonthic appliance for creation of median space for safe osteotomy all the patients were operated on by the same surgeon using the same technique. Under general anaesthesia, subtotal LeFort I osteotomy was performed with an additional median osteotomy of the maxilla and the palate. Osteotomy lines were carried out as in regular LeFort I with complete disjunction in the pterygo-maxillary fissure; the only difference was leaving the posterior aspect of the lateral nasal wall around descending palatine artery intact [20]. A bone-borne palatal distractor (DePuy Synthes) [21] (Fig. 1) was activated after 1 week with a daily distraction of 0.33 mm. The distraction was carried out until desired expansion was achieved. In regular check-ups the distance between left/right canine and first molar teeth was measured until the planned distances were achieved. When the planned expansion was achieved, the distractor was kept in place for 4 months as a rigid retention. An active orthodontic treatment with a fixed appliance (braces and wires) was initiated 8–10 weeks after the expansion to eliminate the diastemas, align the dental arch, and achieve a good occlusion.

**Fig. 2:**
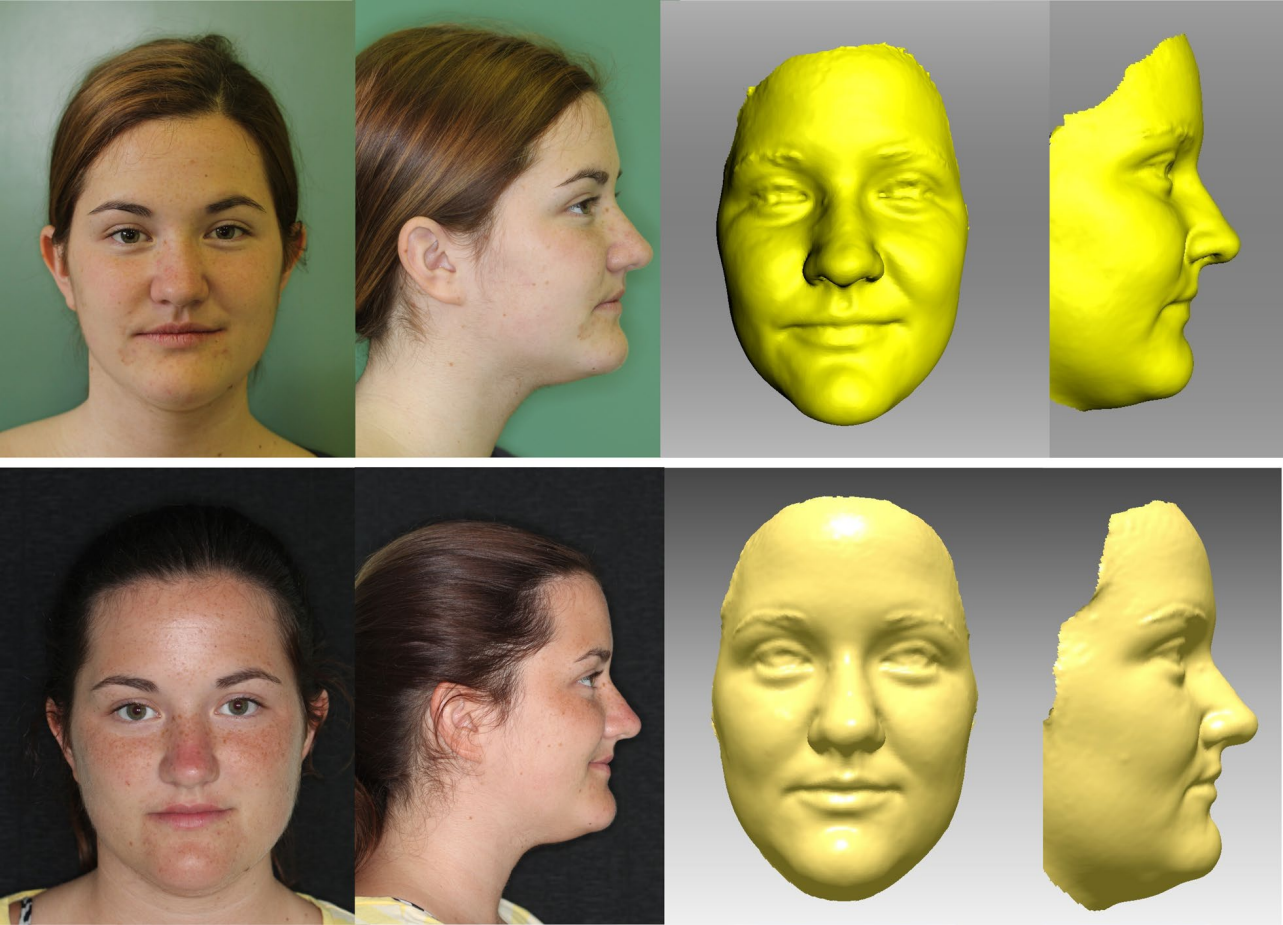
Clinical photographs and 3D scans of the patients before surgery (upper raw) and 6 months after surgery (lower raw) showing slight changes in the area of upper lip

**Fig. 3:**
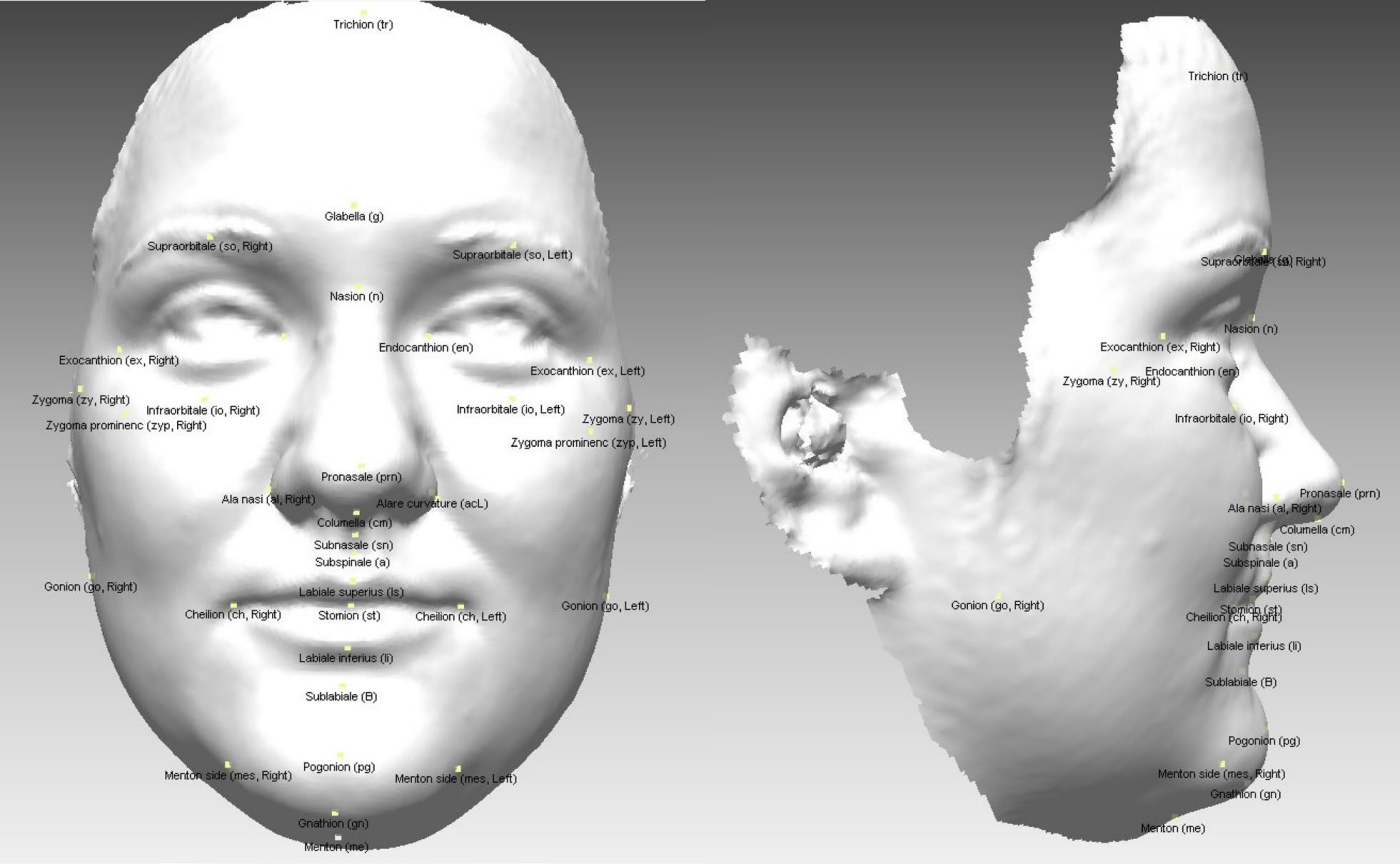
3D facial image with anthropometric landmarks. En face view on the left side and profile view on the right side. In picture 1 presented inner eye distance (enL-enR), outer eye distance (exL-exR), nasal ala width (alL-alR), facial width (zyL-zyR), gonion width (goL-goR), mouth width (chL-chR), intermental width (meL-meR); in picture 2 face height (g-pg), upper face height (g-sn), lower face height (sn-pg); in picture 3 nasal dorsum length (n-prn), nasal tip protrusion (prn-sn), vertical philtrum height (sn-st); in picture 4 facial profile angle (n-sn-pg) and in picture 5 nasolabial angle (cm-sn-ls) and nasal width angle (acL-prn-acR)

In addition to the standard diagnostics, two 3D facial surface scans of each patient were obtained with a 3D optical scanner (Artec 3D, Artec MHT [22]). The scans were taken in a relaxed environment, with the subjects sitting on an ergonomic chair in a straightened posture and with patient’s head in its natural position [23–25]. The volunteers were asked to refrain from any movements—if possible, as well from blinking—for the period of the scanning procedure (about 15–20 s) with a 3D optical scanner. A pre-operative 3D image was taken on the day of the operation and the second image, 6 months after the operation at the end of the retention period (Fig. 2). The 3D facial images were imported into RapidForm 2006 (INUS Technology, Inc., 601-20, Yeoksam-dong, Gangnam-gu, Seoul 135-080, South Korea) computer software. Thirty-nine (39) 3D anatomical landmarks [26] were identified on the facial surface of each image (Table 1, Fig. 3).

**Table 1:** List of facial landmarks (cephalometric points) used in the analyses and its definiton

Sign	Name	Definition
tr	Trichion	Midpoint between scalp and forehead
g	Glabella	The most anterior midpoint of the forehead
n	Soft tissue nasion	The most posterior midpoint on the soft-tissue contour of the base of the nasal root at the level of the frontonasal suture
enL, enR	Endocanthion, left and right	Soft-tissue point located at the inner commissure of each eye fissure
exL, exN	Exocanthion, left and right	Soft-tissue point located at the outer commissure of each eye fissure
soL, soR	Supraorbitale, left and right	The most anterior point above the orbita (midpoint between endo- and exo-canthion)
ioL, ioR	Infraorbitale, left and right	Prominent rim under the inferior eyelid (midpoint between endo- and exo-canthion)
zyL, zyR	Zygoma, left and right	Point above lateral part of corpus ossis zygomatici before it straightens in the AP direction backwards
zypL, zypR	Zygoma prominenc, left and right	Intersection of vertical line through exL/R and transverse line through zyL/R
prn	Pronasale	The most anterior midpoint of nasal tip
alL, alR	Ala nasi, left and right	The most lateral point on each alar contour
acL, acR	Alare curvature, left and right	Point at the facial insertion of each alar base
cm	Columella	Midpoint of the columella crest at the level of the nostril top points
sn	Subnasale	Contact of philtrum and columella
a	Subspinale	The most posterior midpoint of the philtrum
lsL, lsR	Labiale superior, left and right	Midpoint of the vermilion line of the upper lip
St	Stomion	Midpoint of the horizontal labial fissure
liL, liR	Labiale inferior, left and right	Midpoint of the vermilion line of the lower lip
cphL, cphR	Crista philtri, left and right	Point at each crossing of the philtrum and cupids bow
chL, chR	Cheilion, left and right	Point at each labial commissure
b	Sublabiale	The most posterior midpoint on the labiomental soft-tissue contour that defines the border between the lower lip and the chin
pg	Pogonion	The most anterior midpoint of the chin
gn	Gnathion	The midpoint between pogonion and menton
me	Menton	The most inferior midpoint of the chin
mesL, mesR	Menton side, left and right	The point where the vertical point through chL/R reaches the lowest point of the chin
goL, goR	Gonion, left and right	Ramus ascendens and corpus mandible tangents intersection

**Fig. 4:**
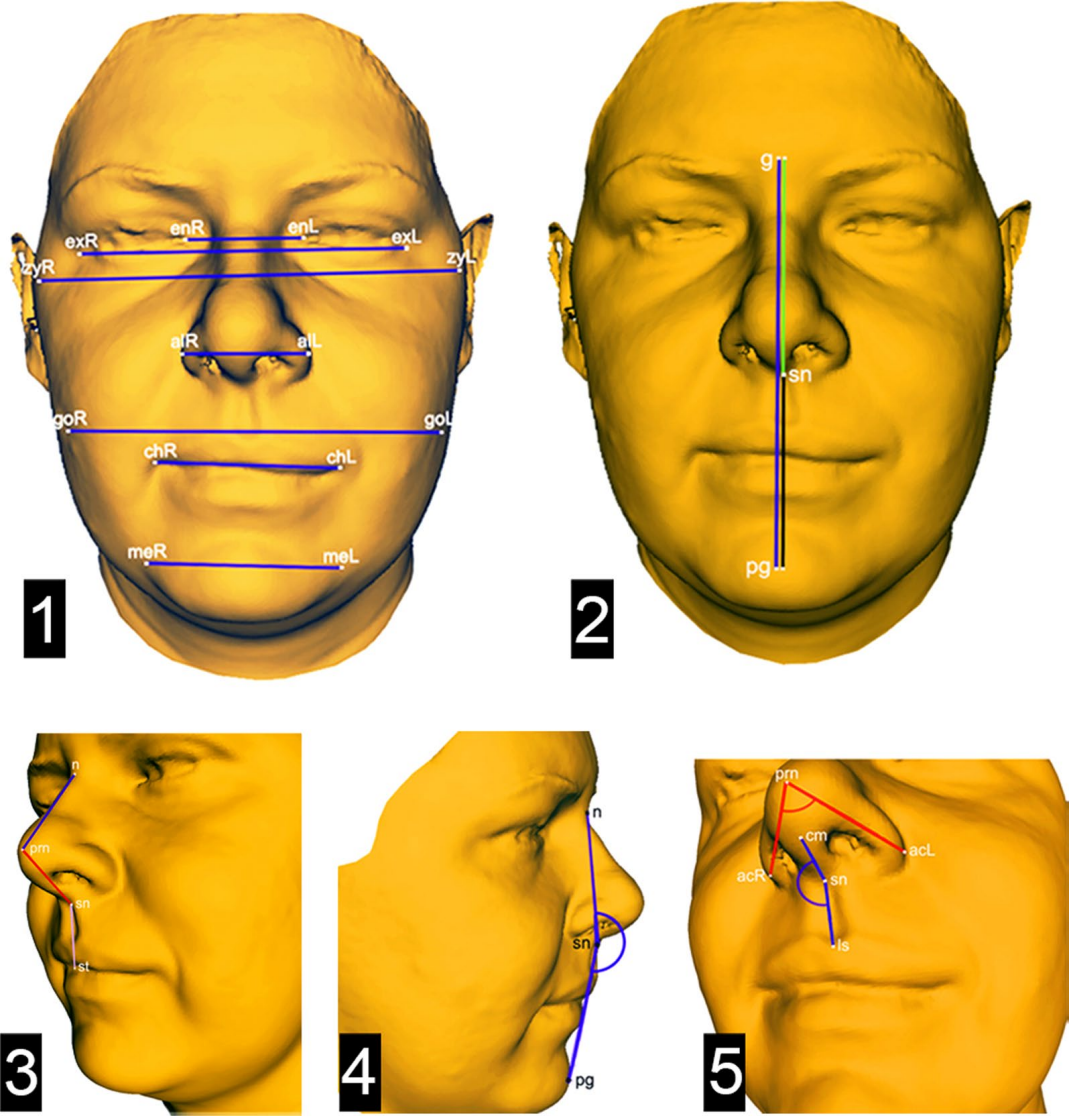
3D facial image with selected parameters in the transverse (1), vertical (2, 3), and sagittal planes (4, 5)

Before the study, the intra-rater reliability was verified with an intraclass correlation and we confirmed that the method is reliable. Using the coordinates (x, y, z) of identified landmarks (Fig. 3) we were able to calculate the linear distance between the points and measure the angles. Various facial parameters in the transverse and vertical directions and also the angles were chosen to anthropometrically (cephalometric analysis of the 3D facial scan) analyse the facial morphology (Table 2, Fig. 4).

**Table 2:** List of observed parameters used in the anthropometric analyses and its definiton

Parameter	
Inner eye distance | enL-enR (mm)	The distance between the left and right endocanthions
Nasal ala width | alL-alR (mm)	The distance between the left and right alae nasi points
Nasal base width | acL-acR (mm)	The distance between the left and right alae nasi curvature points
Facial width | zyL-zyR (mm)	The distance between the left and right zygoma
Gonion width | goL-goR (mm)	The distance between the left and right gonion
Mouth width | chL-chR (mm)	The distance between the left and right cheilions (the point at each labial commisure)
Intermental width | meL-meR (mm)	The distance between the left and right menton
Face height | g-pg (mm)	The distance between the glabella and pogonion points
Upper face height | g-sn (mm)	The distance between the glabella and sub-nasale points
Lower face height | sn-pg (mm)	The distance between the sub-nasale and pogonion points
Nasal dorsum length | n-prn (mm)	The distance between the base of the nose (nasion point) and the tip of the nose (pronasale point)
Nasal tip protrusion | prn-sn (mm)	The distance between the pronasale and subnasale points
prn-pg (mm)	The distance between the pronasale and pogonion points
Vertical philtrum height | sn-st (mm)	The distance between the sub-nasale and stomion points
Facial profile angle | n-sn-pg (°)	The angle between the nasion, sub-nasale and pogonion points
Nasolabial angle | cm-sn-ls (°)	The angle between the collumela, subnasale and the upper lip
Nasal width angle | acL-prn-acR (°)	The angle between the alare curvature, pronasale and the alare curvature on the other side

**Fig. 5:**
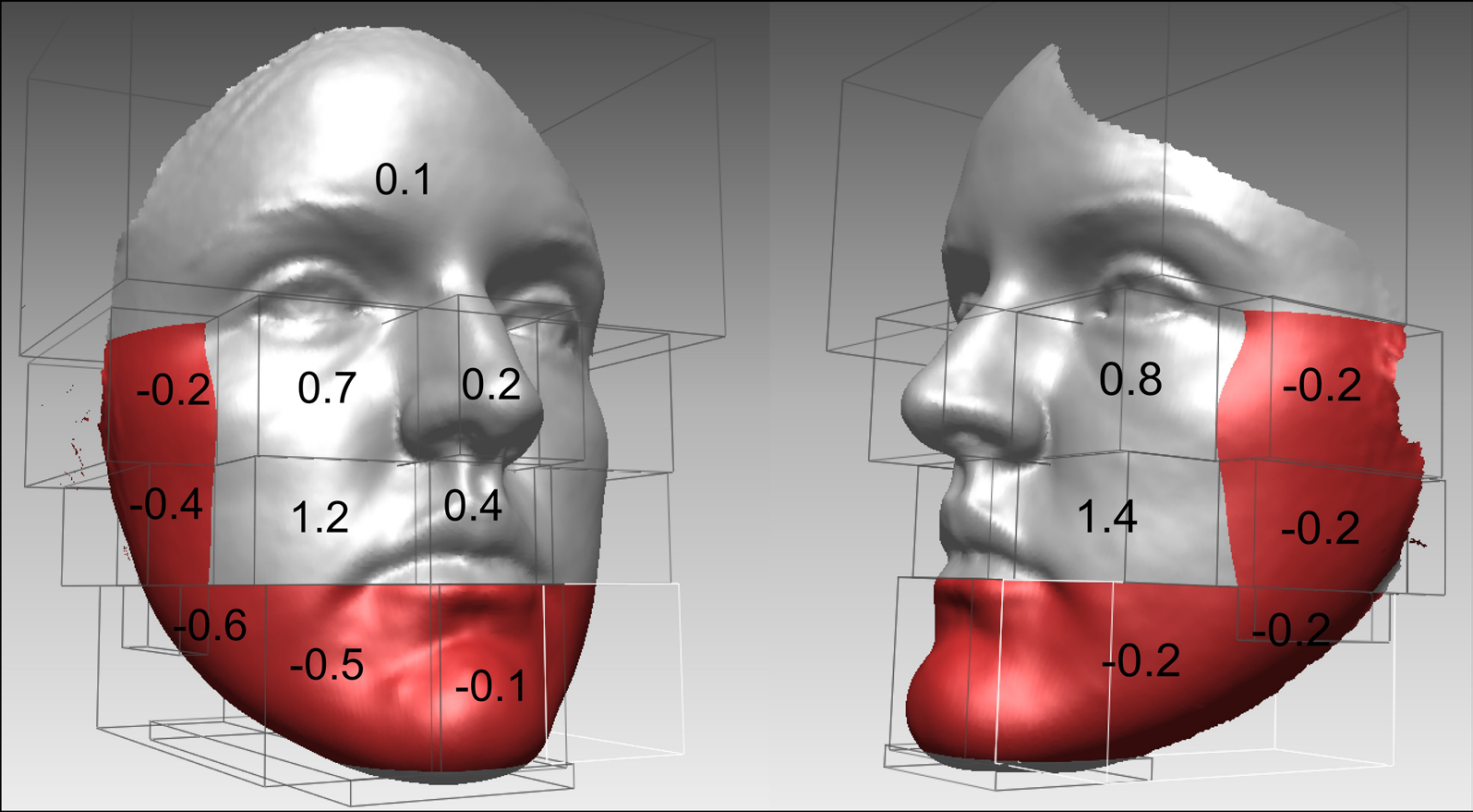
Left—Facial surface divided into five main areas (1–5) using four horizontal planes. Four vertical planes additionally subdivide areas 2–4 into the central, medial and lateral regions (r-right, c-central, l-left, M-medial, L-lateral. Middle—Color map of the superimposed 3D scans, and color histogram on the right, demonstrating the degree of deviation between the pre- and post-operation facial images of one selected patient. The increased blue saturation corresponds to the positive distance, which is most intensively seen in the paranasal area and in the area superior and lateral to the upper lip and the red saturation corresponds to backward changes

A facial surface-areas comparison was additionally conducted. Both 3D scans of each patient were digitally registered in the same workplace using the regional best-fit method. The forehead, supraorbital and nasal root regions were selected for the superimposition. Four vertical and four horizontal planes were used to divide the facial surface into 17 areas (Fig. 5). A software tool was then able to calculate the average difference in the displacement between both 3D images in each region (shell-to-shell difference) corresponding to the soft-tissue changes following the treatment.

All the acquired data from the anthropometric facial analysis and the facial surface-areas comparison were imported to Microsoft Excel and IBM SPSS Statistics for statistical analyses. The pre- and post-expansion facial parameters were compared with the paired t test. The significance level was set at p < 0.05. A multivariate linear-regression model was later used to correlate the maxillary expansion width (the amount of distractor expansion in mm) to the soft-tissue changes found in the observed regions. Because the changes on the left- and right-hand sides of the face showed similar values, we merged them and compared them as a common lateral area.

## Results

All included participants in this cohort observational study had been scanned before surgery and 6 months after. An anthropometric (3D cephalometric) analysis was performed on the facial images before and after the maxillary expansion and the results were compared (Table 3). In the cephalometric analysis, significant differences occurred for an increased nasal width (at the level of the nasal ala for 1.1 mm and the nasal base for 2.2 mm), a decreased upper-face height (for 1.4 mm) with an unchanged lower facial height, an increased vertical philtrum height (for 0.8 mm) and an increased nasolabial angle (for almost 3 degrees). A significant increase in the facial profile angle was also observed, resulting in an increased facial convexity and anterior displacement of the upper lip area.

**Table 3:** Anthropometric analysis results showing the values (mean ± standard deviation) of the observed parameters before and after the operation, their differences (with 95% confidence interval) and their comparison with the t test (p value)

Parameter	Before	After	Diff. (95% CI)	Sig
Inner eye distance | enL-enR (mm)	32.5 ± 3.3	32.5 ± 2.9	0.0 ± 1.3 (− 0.8; 0.7)	0.962
Nasal ala width | alL-alR (mm)	35.0 ± 1.9	36.2 ± 2.2	− 1.1 ± 1.7 (− 3.2; − 1.3)	0.019*
Nasal base width | acL-acR (mm)	33.4 ± 2.1	35.6 ± 2.4	− 2.2 ± 1.7 (− 5.3; − 3.5)	< .001*
Facial width | zyL-zyR (mm)	135 ± 7.0	134.0 ± 6.0	0.7 ± 2.8 (− 0.2; 2.9)	0.380
Gonion width | goL-goR (mm)	126 ± 6.0	125.0 ± 6.0	1.3 ± 2.8 (1.0; 4.1)	0.095
Mouth width | chL-chR (mm)	48.2 ± 5.6	48.5 ± 5.1	− 0.2 ± 2.8 (− 2.0; 1.1)	0.758
Intermental width | meL-meR (mm)	57.1 ± 4.7	56.2 ± 4.3	0.9 ± 4.4 (− 0.7; 4.2)	0.454
Face height | g-pg (mm)	130.0 ± 9.0	129.0 ± 10.0	1.4 ± 2.9 (1.2; 4.5)	0.082
Upper face height | g-sn (mm)	72.4 ± 5.0	71.0 ± 5.0	1.4 ± 1.7 (1.9; 3.8)	0.006*
Lower face height | sn-pg (mm)	58.9 ± 6.7	59.0 ± 7.0	− 0.1 ± 3.1 (− 2.0; 1.4)	0.857
Nasal dorsum length | n-prn (mm)	50.7 ± 4.9	50.5 ± 4.3	0.2 ± 1.6 (− 0.6; 1.3)	0.688
Nasal tip protrusion | prn-sn (mm)	23.1 ± 1.9	22.5 ± 2.2	0.6 ± 1.3 (0.5; 2.0)	0.092
prn-pg (mm)	76.6 ± 7.3	76.4 ± 7.5	0.2 ± 2.7 (− 1.1; 1.9)	0.802
Vertical philtrum height | sn-st (Mm)	21.6 ± 2.7	22.4 ± 2.9	− 0.8 ± 1.2 (− 2.3; − 0.9)	0.025*
Facial profile angle | n-sn-pg (°)	193.0 ± 8.0	195.0 ± 8.0	− 1.7 ± 2.7 (1.9; 5.0)	0.032*
Nasolabial angle | cm-sn-ls (°)	111.0 ± 7.0	114.0 ± 7.0	− 2.9 ± 3.9 (3.6; 8.0)	0.013*
Nasal width angle | acL-prn-acR (°)	57.9 ± 4.7	64.2 ± 7.0	− 6.3 ± 4.9 (− 15.3; − 9.9)	< .001*

The statistically significant differences are marked with an *

**Fig. 6:**
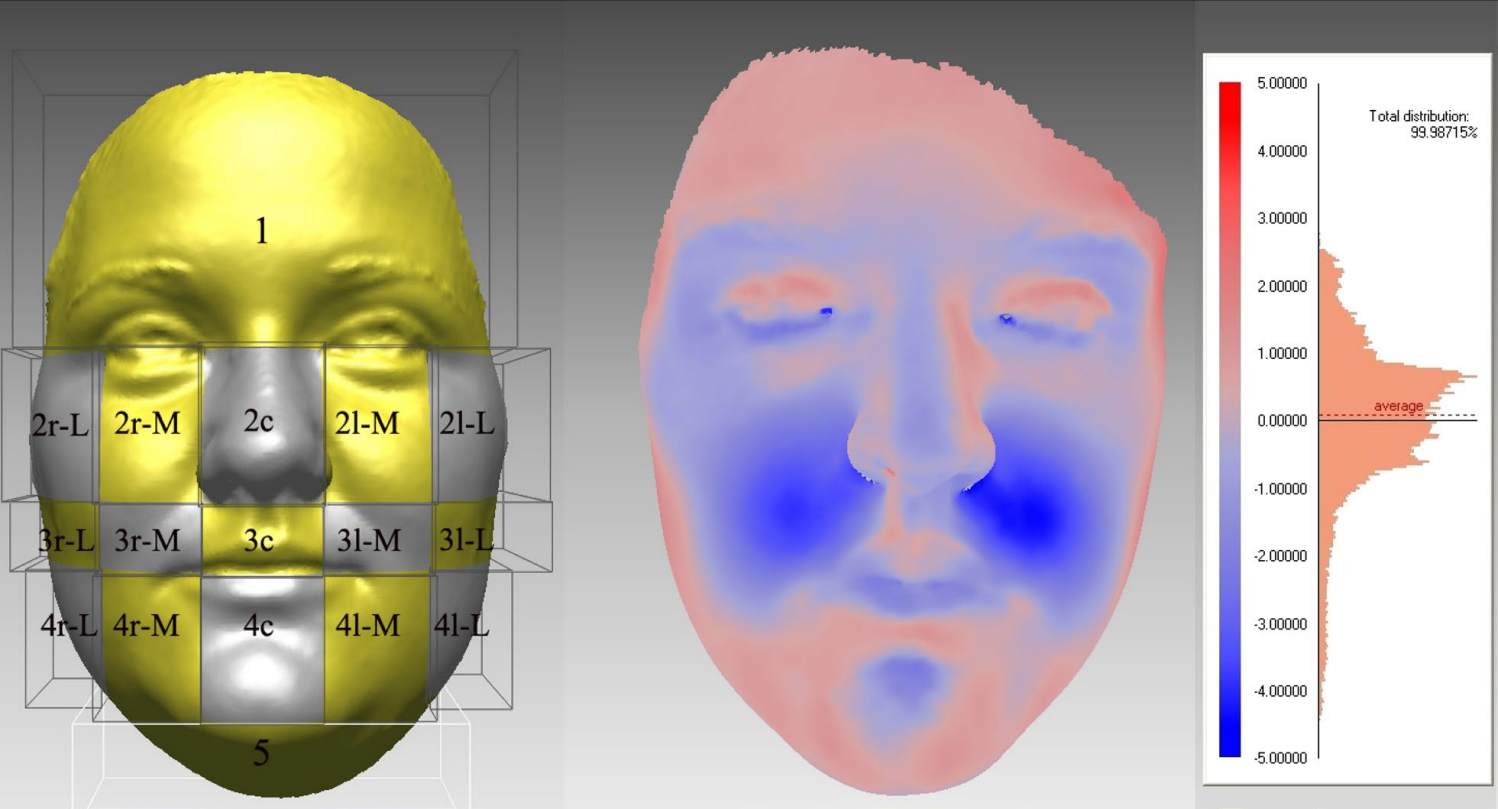
The mean facial differences between the pre- and post-operative scan in the observed regions in mm (the value presents the sum of displacement vectors for all the points in observed region). The red color presents the areas with backward changes and the grey color the forward changes

To include all the surfaces and the data of the 3D image a shell-to-shell comparison was performed, presented with a color histogram in Fig. 5. Figure 6 presents the average distance between the pre- and post-operative scans in the selected areas and the results are presented in Table 4. The greatest differences in the displacements between the pre- and post-operation scans were observed for the paranasal and cheek area (1.4 ± 1.0 mm) in the lateral direction.

**Table 4:** Average distances between pre- and post-operative scans in different facial areas (Fig. 5) presented as the mean values (together with one standard deviation) in selected regions

Region	Average distance (mm)				
	Right lateral	Right medial	Middle	Left medial	Left lateral
2	− 0.2 ± 0.9	0.7 ± 0.9	0.2 ± 0.8	0.8 ± 0.9	− 0.2 ± 0.9
3	− 0.4 ± 0.9	1.2 ± 1.0	0.4 ± 1.0	1.4 ± 1.0	− 0.2 ± 0.9
4	− 0.6 ± 0.5	− 0.5 ± 1.0	− 0.1 ± 0.9	− 0.2 ± 0.8	− 0.2 ± 0.5

The soft-to-hard tissue ratio was used to correlate the soft-tissue changes with underlying bone movements. The results of the linear-regression model did not show any statistically significant correlations between the amount of the maxillary expansion and the observed soft-tissue changes. However not significant the highest coefficient values were observed in areas 3C (0.41, significance 0.17) and 3 M (0.22, significance 0.33).

## Discussion

Due to good orthodontic primary care and due to calculated number of patient needed, the resultant sample is minimal. 3D imaging with stereometric surface scanners is a frequently used method for a facial soft-tissue evaluation in orthognathic surgery with an already-proven high level of accuracy and reliability [27]. There are many factors that could influence 3D facial image; aging changes [28], mimicry [29] but with standardized conditions and within the chosen time limit those factors should not statistically influence the results. We managed to achived standard condition for all the patients; one person operated, one person was scanning, the same time intervals was used. The linear-regression model did not manage to obtain any significant results, probably because of the relatively small variance in the maxillary expansion width, and, especially, because of the smaller sample. For more accurate evaluation of hard to soft tissue ratios more precise methods for bone movements, such as CBCT would be needed.

The anthropometric analysis of the pre- and post-expansion 3D facial scans demonstrated a statistically significant widening of the nose after expansion at the level of the nasal ala (+ 1.1 mm) and the nasal base (+ 2.2 mm). The consequence of a wider nasal base was a statistically significant increase in the nasal width angle (AcR-Prn-AcL). The width of the mouth remained almost unchanged. It is primarily determined by the orbicularis oris muscle, which lacks the direct origin or insertion with a bony jaw. This could be the reason why the SARME does not seem to have a significant influence on the width of the mouth.

The vertical dimension demonstrated two significant changes. The upper-face-height parameter significantly decreased, indicating the sub-nasale landmark, and the surrounding tissues moved in the superior direction (towards the glabella landmark), while the lower-face height remained unchanged. This superior positioning of the sub-nasale is probably one of the consequences of the complex effect SARME has on the nose morphology, including widening. Conflicting findings were reported by Lagravere et al. [30], who concluded that vertical and sagittal skeletal changes following SARME are not clinically significant. The lower-face height remained unchanged, which suggests that no mandibular rotation occurs. In a study by Tong et al. [31] a displacement of the chin (pogonion) in a posterior and inferior direction was demonstrated, suggesting the clockwise rotation of the mandible can occur. Also, Oliveira et al. [32] reported in their study using CBCT (cone beam computer tomography) scans that a clockwise rotation of the mandible is a transient effect immediately after SARME, but tends to return close to the initial values 6 months after expansion. Secondly, the vertical philtrum height significantly increased, as a result of superior positioning of the sub-nasale area. Contradictory findings were reported by Alves et al. [11], who concluded that nasal length and projection and upper-lip length (philtrum height in our study) were not altered by SARME**.**

A significant increase in the soft-tissue facial profile angle also demonstrates changes in the sagittal direction after SARME. Since the expansion occurs in the middle third of the face, it is clear that anterior repositioning of the sub-nasale landmark occurs. The analysis indeed demonstrated anterior positioning of this area (3C, upper lip area). As a result, the facial profile increased in convexity following the SARME treatment. This is important to consider in treatment planning since some MTD patients also exhibit simultaneous sagittal dento-facial deformity, such as skeletal Class II or III [2]. These findings suggest that SARME tends to improve the skeletal Class III malocclusion what was shown also with CBCT after treatment with microimplant-assisted RPE in Asian sample [33]. Our results contradict the results of Nada et al. [18], who found slight retro-positioning of the central part of the upper lip in both groups. Even though our method of 3D facial surface imaging cannot assess bone movements directly, we can still presume that some degree of maxillary advancement occured following SARME in our patients. This difference could be attributed to the surgical technique, since the posterior part of the lateral nasal wall remained intact in our subjects. Therefore, the forces of the trans-palatal distractor, besides the transversal expansion, probably move the maxilla slightly anteriorly. Another important sagittal effect encountered in the presented study was a significant increase in the nasolabial angle.

Regional analysis (Figs. 5, 6) showed the small changes after SARME over the whole face. They are the largest immediately over the expanded areas, but also occur in the lower third of the face, below the lower jaw and in posterior parts of the face. It was observed that widening of the soft tissues over expanded areas consequently narrows the posterior and lower parts of the face. This study objectively evaluated that the facial soft-tissue mask acts as one unit, as already reported in other orthognathic procedures [34].

## Conclusion

Our study objectively evaluates facial changes following SARME in all three dimensions. However, not only do some of our results differ from those of other studies, but the methodology and 3D observation of the whole face also seem to be different. We have confirmed some of the already-known facts, such as widening of the nose and increased projection in the cheek. On the other hand, an increased facial convexity after expansion is a new finding, reflecting the underlying advancement of the maxilla. Furthermore, we observed a reduced upper-face height, while the lower height remained unaffected.

## Data Availability

The datasets used and/or analysed during the current study are available from the corresponding author on reasonable request.

## Abbreviations

3D: Three-dimensional
SARME: Surgically assisted rapid maxillary expansion
MTD: Maxillary transverse deficiency
CBCT: Cone beam computer tomography
